# Vertical Fracture of the Medial Malleolus With Concurrent Ipsilateral Achilles Tendon Rupture in a Patient With Progressive Collapsing Foot Deformity: A Rehabilitation Dilemma

**DOI:** 10.7759/cureus.57750

**Published:** 2024-04-07

**Authors:** Hannah L Morley, Bawan Hama, Gary Hannant

**Affiliations:** 1 Trauma and Orthopaedics, Bradford Teaching Hospitals NHS Foundation Trust, Bradford, GBR

**Keywords:** fracture management, open reduction and internal fixation, functional rehabilitation, achilles tendon rupture, medial malleolus fracture

## Abstract

A male in his 40s presented with a vertical shear type medial malleolar fracture with an ipsilateral Achilles tendon rupture with a pes planovalgus deformity. Both injuries were diagnosed concurrently on presentation. This is a rare injury pattern with no consensus on optimum management. This is the first case report where pes planus is also described alongside the injuries.

The medial malleolar fracture was successfully treated surgically with an open reduction and internal fixation (ORIF) with antiglide plating. Following ORIF, the patient underwent functional rehabilitation for the Achilles tendon rupture.

The purpose of this case report is to highlight this infrequent injury pattern, which poses a great therapeutic dilemma. The therapeutic considerations regarding surgical and non-surgical approaches to management are thoroughly presented and discussed.

## Introduction

Medial malleolus fractures combined with Achilles tendon ruptures are rare entities. There are few, sporadic case reports in the literature [1-7]. There is one published case series dating back to the 1970s detailing this injury pattern in skiers, but otherwise, to the best of our knowledge, no research exists. There is no internationally recognized consensus regarding the optimal treatment of such injury. Indeed, published case reports have either failed to define the management [1], elected to operate on the Achilles rupture but not the medial malleolus [2], or managed the cases non-operatively [3].

Additionally, the surgical planning in some cases is at the surgeon's preference, as delay in diagnosis is a common problem [1,3,5].

It is important to acknowledge the impact of functional rehabilitation on the management of Achilles tendon injuries [8]. First described in the early 1980s, this technique has grown in popularity since several randomized control trials were published in the early 2000s [9,10]. Various case reports regarding this injury pattern were published prior to functional Achilles rehabilitation being in regular use, and this may explain why the Achilles tendon is often reported as being managed operatively.

## Case presentation

An otherwise healthy male in his 40s presented to the emergency department following an uncontrolled fall while walking downstairs. He was unable to tolerate weight-bearing on his injured lower extremity. On examination, a palpable gap was present at the Achilles tendon, and a Simmonds-Thompson test was positive. The injury was isolated and closed. The patient had no neurovascular abnormalities on presentation. The patient had bilateral pes planus deformities.

**Figure 1:**
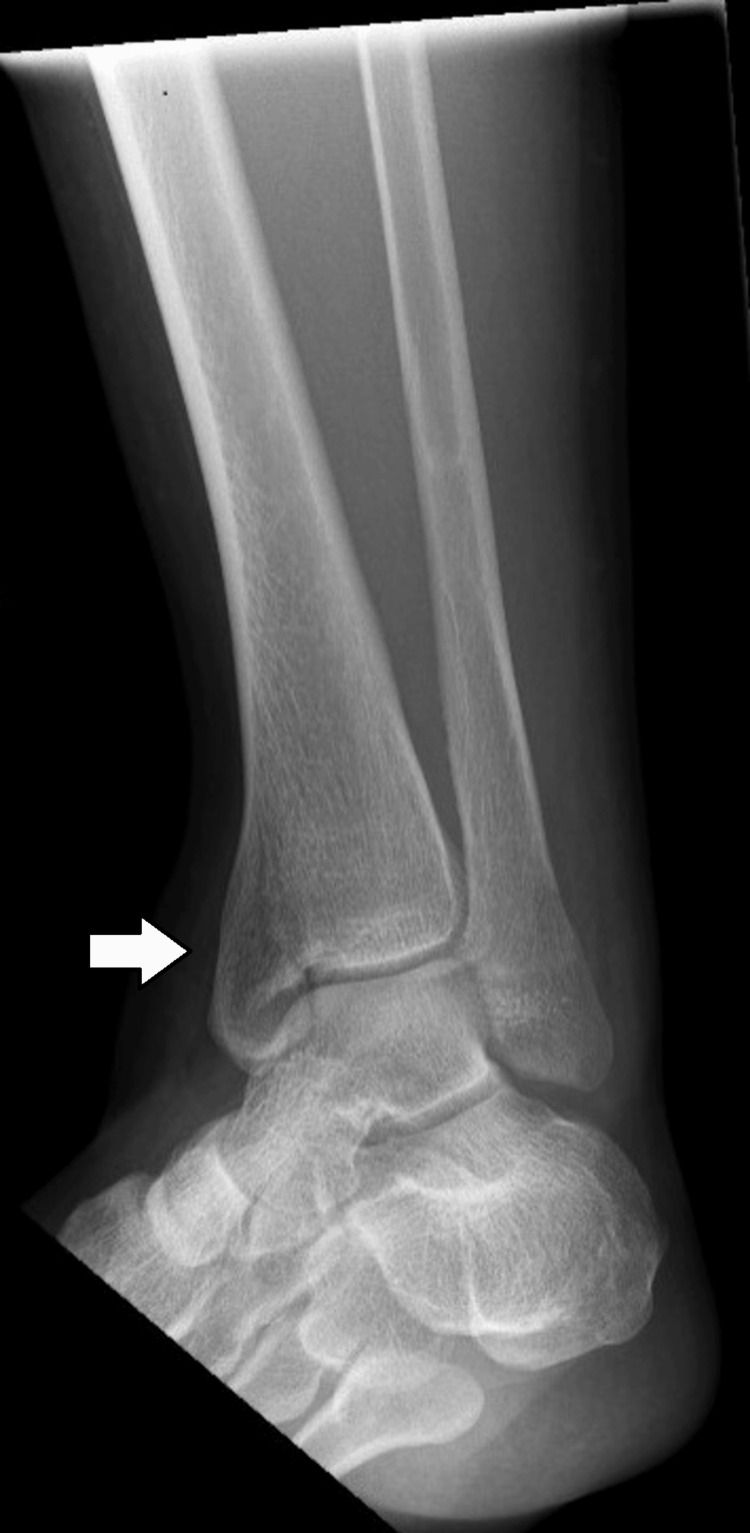
Rotated PA view of the left ankle on admission to the emergency department. The white arrow on the radiograph indicates the medial malleolar fracture. PA: posteroanterior

**Figure 2:**
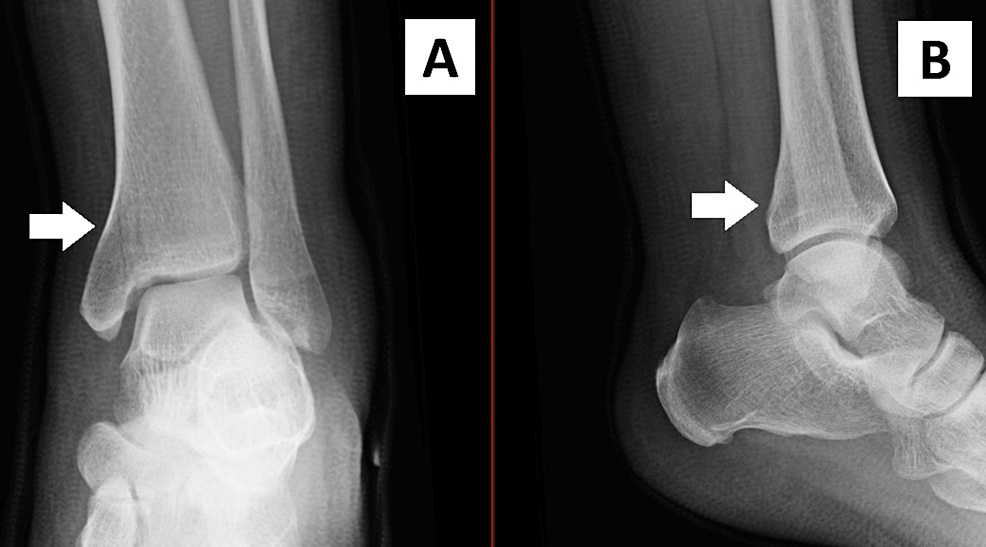
AP (A) and lateral (B) views demonstrating a nondisplaced medial malleolus fracture of the left ankle. The white arrows indicate the location of the fracture. AP: anteroposterior

A clinical diagnosis of an Achilles tendon rupture was made. Plain radiographs of the patient's ankle were performed. A medial malleolus fracture on the ipsilateral leg was diagnosed. These radiographs are shown in Figure 1 and Figure 2. The patient was reevaluated in the fracture clinic the following day.

**Figure 3:**
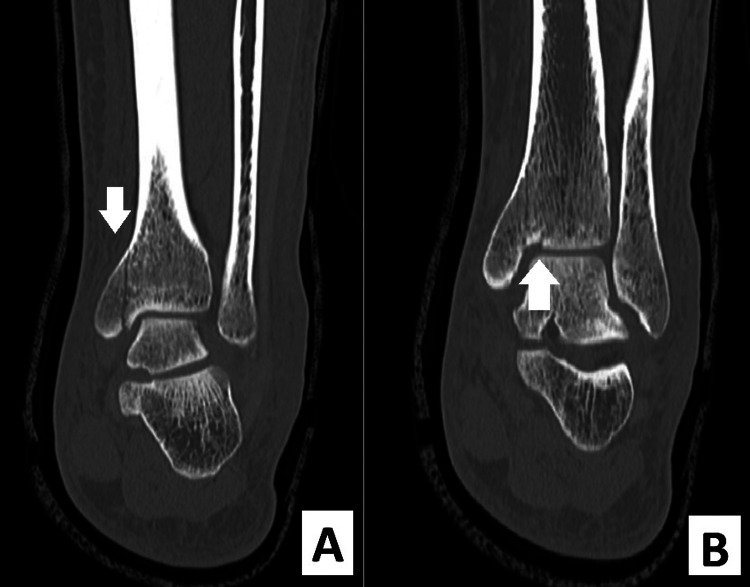
Coronal CT views of the left ankle demonstrating the medial malleolar fracture with comminution into the tibial plafond. A and B demonstrate different coronal sections of the CT scan; this illustrates the fracture's relationship with the joint surface. The arrows indicate the location of the fracture. CT: computed tomography

**Figure 4:**
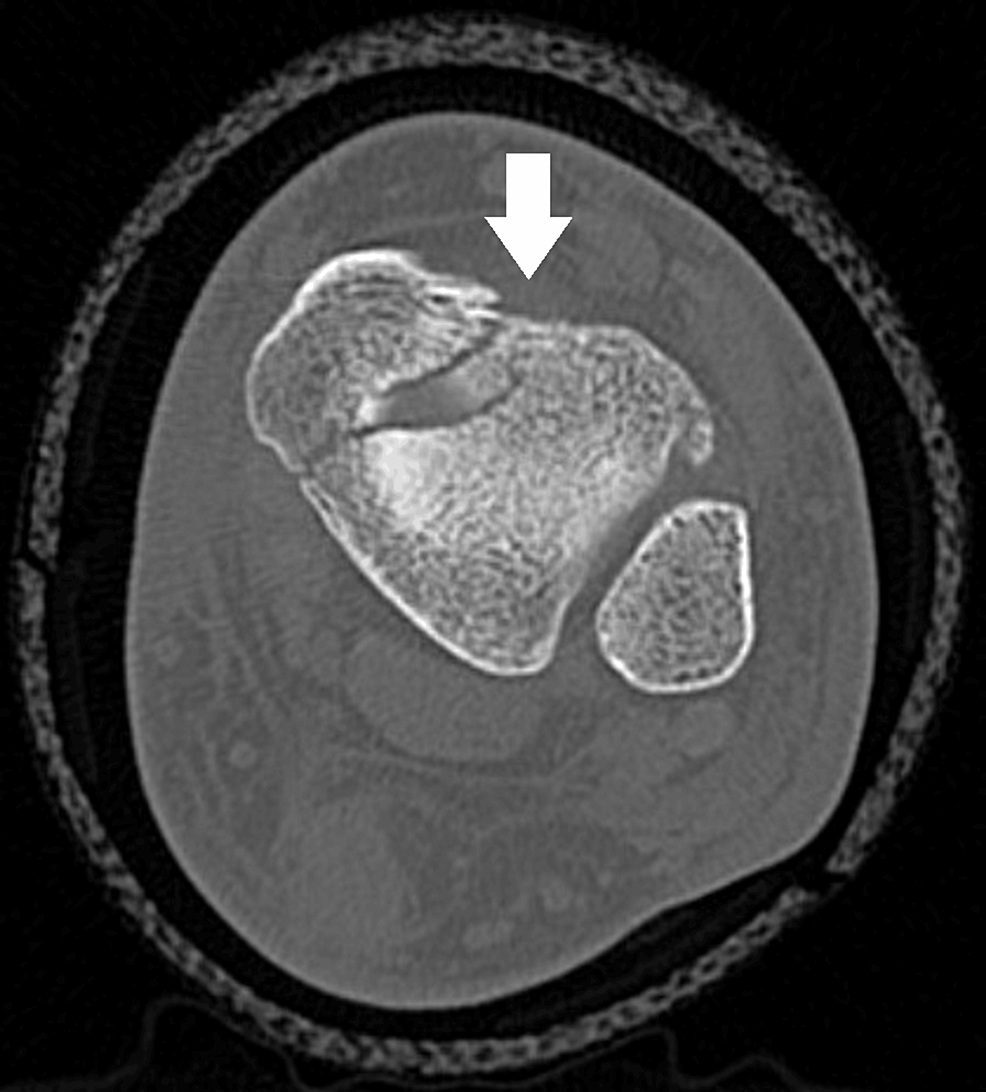
Axial view on CT scan of the medial malleolar fracture with an arrow indicating the location of the fracture. CT: computed tomography

An ultrasound demonstrated a full-thickness tear of the Achilles tendon with a gap of approximately 4.5 cm, 3 cm proximal to the insertion. A computed tomography (CT) of the ankle demonstrated a comminuted intra-articular fracture of the left distal tibia through the medial malleolus. There was also a 1-2 mm step at the articular surface of the tibial plafond. This is best visualized in Figure 3, as well as Figure 4. Although comminution was present on the CT, the fracture fragments remained nondisplaced. Furthermore, there was no posterior malleolus fracture or evidence of disruption of the syndesmosis on the CT scan.

**Figure 5:**
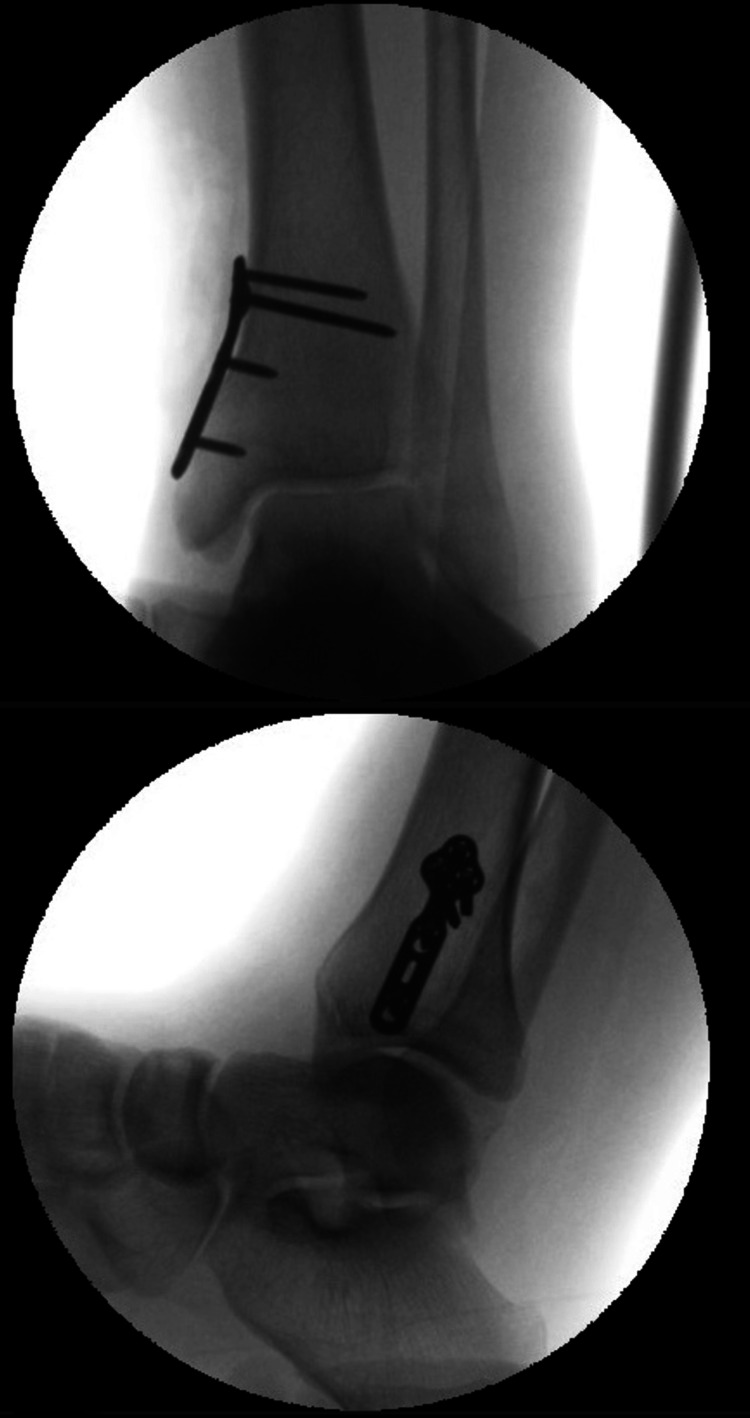
Intraoperative films demonstrating the antiglide plating.

Surgery was performed eight days post-injury to account for soft tissue swelling. The patient was treated with an open reduction and internal fixation (ORIF) of the medial malleolus; this was performed under fluoroscopy and anesthesia. The vertical medial malleolar fracture was fixed with a 2.7 mm four-hole Synthes Variable Angle Cloverleaf Plate [11], in antiglide mode. Once the wounds were closed, the patient was placed in a front slab in equinus in theater to prevent dorsiflexion. Figure 5 demonstrates the intraoperative radiographs. 

The patient had functional rehabilitation using a Vacoped boot system [12] at one week postoperatively. Prophylaxis for venous thromboembolism was provided until the foot was in plantigrade. An example of our local functional Achilles rehabilitation protocol can be found in the Appendices [13].

**Figure 6:**
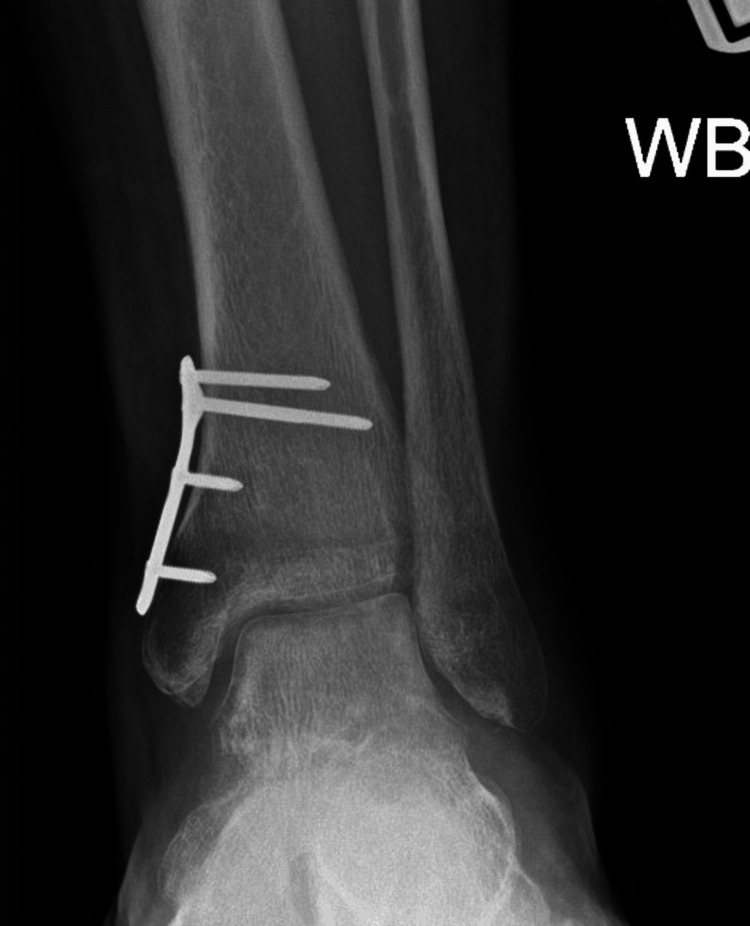
Postoperative weight-bearing AP ankle view. AP: anteroposterior, WB: weight-bearing

**Figure 7:**
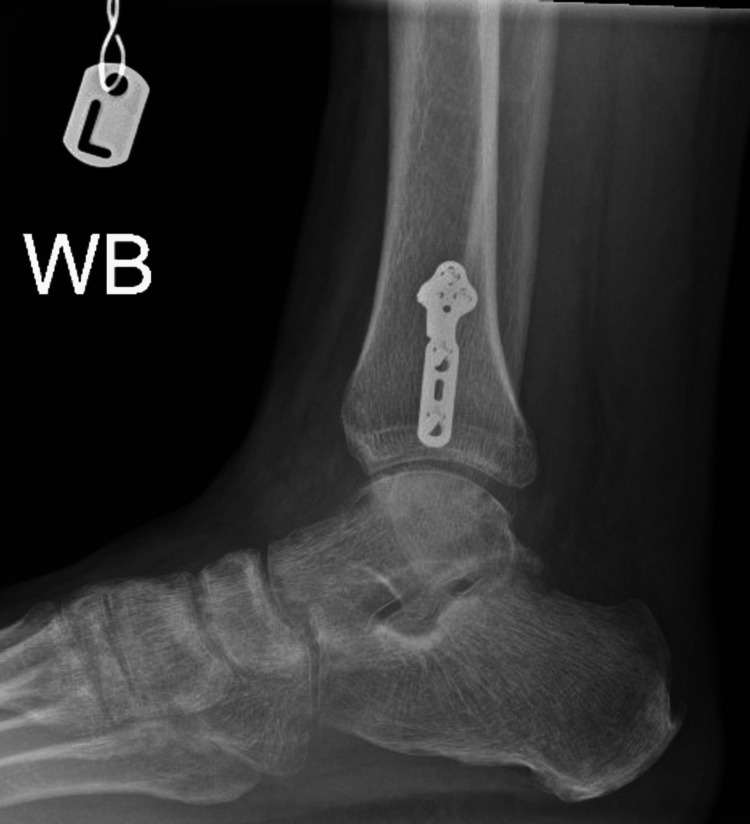
Weight-bearing lateral view of the ankle. WB: weight-bearing

The patient was discharged from face-to-face follow-up at 12 months post-injury and was assessed as being able to perform a single heel raise test prior to discharge and has since returned to manual work. Figure 6 and Figure 7 show the healed fracture on radiographs. We can provide a 24-month follow-up at the time of submission of this article.

The patient agreed to have Achilles Tendon Total Rupture Score (ATRS) and European Foot and Ankle Score (EFAS) data collected throughout his care [14,15]. The final ATRS was 84 at 12 months. ATRS has not been validated on patients with concurrent injuries to Achilles tendon ruptures; however, we felt that it was a useful score to use in this case in the absence of another available score. The final EFAS was 12 at 12 months. The change in score is documented in Table 1.

**Table 1:** ATRS and EFAS for the case described at different timepoints during follow-up. ATRS: Achilles Tendon Total Rupture Scores, EFAS: European Foot and Ankle Score

Time in months post-injury	ATRS	EFAS
Pre-morbid (patient answered this upon diagnosis of injury)	100	24
3	24	2
8	68	12
12	84	14
Total score available	100	24

## Discussion

Combined Achilles tendon rupture and ankle fractures are rare occurrences. There are several reports of a vertical medial malleolar fracture associated with Achilles injury. Previous papers have hypothesized that such a fracture pattern results from sudden ankle hyperextension or hindfoot inversion [2,3]. Other authors have noted the lack of syndesmotic injury [2,3], which was also the case here.

With regard to foot and ankle morphology, the patient in this case had a pes planovalgus deformity. To the best of our knowledge, there are no published cases of pes planovalgus deformity associated with an Achilles tendon rupture and a concurrent medial malleolar fracture, thus making this case report unique. Indeed, literature relating to Achilles tendon rupture seems to refer to hyperpronation not being a risk factor for Achilles tendon rupture [16]. Indeed, in the current literature, there is a paucity of evidence to support the association of pes planovalgus deformity on any specific injury pattern [17,18].

Surgical consensus in the literature is lacking [1-7]. Lu et al. [5] described a case where the medial malleolus was fixated, and the Achilles rupture was non-operatively managed. This treatment approach is equivalent to our case. However, the Achilles rupture in the case of Lu et al. [5] was only identified following the medial malleolus fixation. In their case, the Achilles tendon rupture was managed non-operatively in a fixed cast, not with functional rehabilitation. We believe our case to be the first to describe the use of functional rehabilitation, as we are unable to find specific functional rehabilitation protocols used in the case reports in our literature review. In our case, the Achilles tendon healed without the need for surgery.

Some authors describe operatively managing both injuries [6,7]. However, these injuries are unlikely to be able to be accessed via the same incision, and therefore, there are additional risks associated with wound complications and damage to surrounding neurovascular structures. We would advocate fixing the medial malleolus to create a stable scaffold to rehabilitate the Achilles tendon. In consideration of future cases where there may be an indication for operative fixation of the Achilles tendon but with concern regarding wound healing, options could include the use of percutaneous Achilles repair or endoscopic flexor hallucis longus transfer [19], although this is yet to be described in relation to this injury pattern.

EFAS in this patient improved, but improvement in ATRS lagged. Although there is some overlap between the two patient-reported outcome measures (PROMS), the ATRS is specific to the Achilles tendon, and the EFAS is more generalized. Indeed, although this patient did manage to return to work, he was still in some pain 12 months following surgery. It is unknown whether the period away from the rehabilitation program affected these scores.

This is the first case report of this injury type to include patient-reported outcome measures (PROMS). We would advocate the use of PROMS to other surgeons dealing with this combination of injuries. This could be facilitated through a centralized database, such as the British Orthopaedic Foot and Ankle Society (BOFAS) registry [20]. An international database in foot and ankle surgery would facilitate further research into the management of these concurrent injuries.

## Conclusions

Ipsilateral Achilles tendon rupture with medial malleolar fracture is rare but presents an interesting treatment conundrum. We have demonstrated that a mixed operative and non-operative approach is a viable option, supported by PROMS. Although there is currently no internationally recognized consensus on the management of this unique injury pattern, we present the case that there is no requirement to operate on a ruptured Achilles tendon just because the patient is anesthetized for a concurrent injury. The association between pes planovalgus and this injury pattern is unclear and requires further research. For rarer combination injuries, contributing to registry databases is a useful adjunct to publishing case reports and case series.

## Appendices

Bradford Teaching Hospitals NHS Foundation Trust non-operative tendo-Achilles management protocol and functional rehabilitation and physiotherapy guidelines

Post-Injury Week 0-2​​​​​

1. Referred from the ED to the acute ED musculoskeletal (MSK) clinic. Must be seen in the clinic within 1/52.

2. All patients are to have an urgent ultrasound scan (USS).

3. Below knee equinus cast.

4. Non-weight-bearing (NWB) with elbow crutches.

5. Venous thromboembolism (VTE) assessed, and all patients prescribed a 6/52 course of rivaroxaban.

6. Advise elevation and toe exercises.

Post-Injury Week 2-4

1. Return to acute ED MSK clinic at 2/52 to remove equinus cast, apply Vacoped boot, and demonstrate initial exercises.

2. Vacoped boot locked at 30 degrees equinus (30-degree wedge).

3. Progress to weight-bearing as tolerated with elbow crutches.

4. Must be in boot at all times, including sleeping in boot (external heel can be removed at night).

5. Give the patient a copy of the protocol and information leaflet.

6. Referral to physiotherapy Achilles clinic at St. Luke's Hospital (SLH) (runs Monday morning (FAO Damian Buck and Emma Walmsley)).

7. Exercise in boot: isometric plantarflexion against boot, open kinetic chain quadriceps/gluteal muscle strengthening, hip abduction in side lying, and general upper body strengthening as required.

Post-Injury Week 4-7 (Physiotherapy Outpatients)

1. Vacoped boot 15-30 degrees.

2. 30-degree wedge.

3. Weight-bearing as tolerated in boot.

4. Matles test (symmetry allows for continuation of protocol).

5. Ensure that the patient is familiar with inflating/deflating the boot liner.

6. Patient to sleep in boot (external heel can be removed for sleeping).

7. Pre-injury Achilles Tendon Total Rupture Score (retrospective) at physiotherapy.

8. Exercises in boot: isometric plantarflexion against boot, resisted plantarflexion in knee extension and flexion with yellow theraband at five weeks (avoid using the long toe flexors), partial weight-bearing (PWB), closed kinetic chain (CKC) exercises, quads/glutes strengthening in 30 degrees plantarflexion, and active plantarflexion and dorsiflexion to limit of boot.

Post-Injury Week 7-9 (Physiotherapy Outpatients)

1. Vacoped boot 0-30 degrees plantarflexion.

2. Flat wedge.

3. Full weight-bearing in boot.

4. Exercises in boot: static bike with no resistance, seated calf raises in Vacoped, continue range of motion (ROM) exercises to limit of boot, and progress theraband resistance with plantarflexion strengthening.

Post-Injury Week 9-10 (Physiotherapy Outpatients)

1. Vacoped boot unlocked.

2. Can remove boot at night in bed.

3. Full weight-bearing.

Post-Injury Week 10-12 (Physiotherapy Outpatients)

1. Remove boot and go into own shoe (wean off >2 weeks).

2. Heel raise provided by physiotherapy (size appropriate to patient's dorsiflexion ROM).

3. Gait re-education (can use elbow crutches to assist with gait pattern, encouraging toe-off phase if required).

4. Avoid activities that involve extreme dorsiflexion combined with active ankle plantar flexion (risk of re-rupture), e.g., quick stride up an incline.

5. Do not attempt eccentric lowering exercises.

6. Do not attempt resistance plantar flexion exercises that require more than half the patient's body weight (Hutchison protocol, 2015).

7. Avoid stretching gastroc/soleus (risk of lengthening).

8. Basic static balance and proprioception exercises, e.g., one-leg standing.

9. Bilateral supported heel raises (encourage inner range plantarflexion).

10. Progress frequency and volume with plantarflexion strengthening exercises encouraging inner range to achieve full ROM. Monitor for overuse of long toe flexors, for example: progression of theraband resistance, leg press (up to half of body weight), and double bridging with heel raise.

11. Treadmill walking/bike some resistance.

12. Refer to hydrotherapy. Combine with treatment on dry land.

13. Rowing machine.

14. Complete ATRS 1 post-injury (10/52).

Post-Injury Month 3-5 (Physiotherapy Outpatients)

1. Progress loading of heel raise with weight transfer (aiming for single heel raise (consider gastroc and soleus)).

2. Dynamic balance exercise (wobble board and trampette).

3. Gradual increase in walking speed and incline.

4. Cross trainer.

5. Avoid quick changes of pace or jumping down from a height.

6. Basic plyometrics, e.g., double leg jumps once able to perform a single heel raise.

7. Dispense with heel raise.

8. Complete ATRS 2 post-injury (4/12).

Post-Injury Month 5-6 (Physiotherapy Outpatients)

1. Begin jogging at 20 weeks on a flat surface.

2. Gradual progression of sport-specific drills as required, e.g., change of pace/direction, bilateral to unilateral jumps.

3. Commence eccentric strengthening off step: double leg eccentric strengthening into dorsiflexed position, progress to single leg eccentric strengthening into full dorsiflexed position, and increased weight/resistance with strengthening exercises.

4. Progress proprioception exercises, e.g., single-leg landing.

Post-Injury Month 6-8 (Physiotherapy Outpatients)

1. Introduce single-leg hopping with vertical and horizontal progressions.

2. Hill running.

3. Complete ATRS 3 post-injury (8/12).

Post-Injury Month 8+

1. Aiming to return to sport and full activity.

2. Return to sport criteria: controlled single heel raise with limb symmetry index > 90% of the contralateral leg, ability to sprint with toe-off phase of gait, triple hop test at least 90% of the contralateral leg, and vertical hop test at least 90% of the contralateral leg.

One Year Post-Injury

1. Phone call for ATRS 4 post-injury.
